# Red blood cell distribution width as a marker of cerebral infarction in hemodialysis patients

**DOI:** 10.1080/0886022X.2017.1398664

**Published:** 2017-11-16

**Authors:** Liyi Mo, Yuanhan Chen, Zhilian Li, Lixia Xu, Wei Dong, Li Zhang, Huaban Liang, Ruizhao Li, Yiming Tao, Wei Shi, Xinling Liang

**Affiliations:** a Department of Nephrology, Guangdong General Hospital and Guangdong Academy of Medical Sciences, Guangzhou, China;; b Department of Nephrology, Dongguan People's Hospital, Dongguan, China

**Keywords:** Red blood cell distribution width, hemodialysis, risk factor, cerebral infarction, stroke

## Abstract

**Background:** Red blood cell distribution width (RDW) is a cardiovascular biomarker. We evaluated the association between RDW and cerebral stroke risk in hemodialysis patients.

**Methods:** A cohort of 442 adult patients on hemodialysis was studied. Strokes were defined according to ICD-10 diagnosis codes. Routine complete blood counts, evaluated every 3–6 months, were used for RDW values.

**Results:** Among 442 hemodialysis patients, during the 50-month follow-up, there were 62 cases (14.0%) of cerebral stroke: 41 (9.3%) with cerebral infarction and 21 (4.8%) with cerebral hemorrhage. Compared with nonstroke patients, a significantly higher RDW was measured in patients with cerebral stroke and cerebral infarction. However, no significant difference was seen in RDW between patients with cerebral hemorrhage and nonstroke patients. After adjustment by age, hypertension, albumin, Charlson Comorbidity Score, and C-reactive protein in different multivariable Cox regression models, patients with the highest mean RDW quartile had a 2.55-fold (hazard ratio = 3.55; 95% confidence interval: 1.33–9.51) higher risk of developing cerebral infarction relative to those with the lowest mean RDW quartile. RDW was not an independent risk factor for cerebral hemorrhage.

**Conclusions:** Increased RDW is an independent risk factor of cerebral infarction in hemodialysis patients.

## Introduction

Cerebral stroke is the second most common cause of death worldwide. Patients with reduced renal failure are at elevated risk of stroke [1], especially in those undergoing hemodialysis [2]. It has been estimated that hemodialysis patients have a 10-fold higher incidence of cerebral stroke than that of the general population [3]. A multicenter cohort study reported a prevalence of cerebral stroke after hemodialysis of 8.3% in the largest dialysis facilities in China [4], which accounted for 20.3% of deaths [5]. In addition to the conventional risk factors for stroke, such as hypertension, diabetes mellitus (DM), certain nontraditional biomarkers (e.g., inflammation, oxidative stress), are found to correlate with stroke in dialysis patients [3]. Identification of markers targeting the risk factors for stroke is, therefore, of great importance to screen the high-risk population.

As a parameter measured in a conventional complete blood count, red blood cell distribution width (RDW) describes the variation in red blood cell (RBC) volume. Mathematically, it is calculated as: RDW = standard deviation (SD) of mean corpuscular volume (MCV)/mean MCV × 100. The presence of more immature RBCs in peripheral blood suggests worse hematopoietic function in the bone marrow and more severe destruction of RBCs [6].

The complete blood count is a routine measurement in hemodialysis patients. RDW has become a required measurement, but the importance of this parameter is often underestimated. Previously, RDW had been regarded only as a marker of iron deficiency in hemodialysis patients [7]. Recently, it was found to correlate with a poor prognosis of cardiovascular diseases (CVDs) [8,9], notably ischemic heart disease [10,11]. Two recent studies that assessed the associations of RDW and CVD in dialysis patients showed that increasing RDW was a risk factor for adverse CVD outcomes [12]. RDW is correlated with oxidative stress [13], which triggers the atherosclerosis responsible for stroke. In addition, RDW was found to have a relationship with intima media thickness of carotid arteries [14,15], suggesting that it may be a marker for cerebral stroke. However, little is known about the association between cerebral stroke, as an endpoint, and RDW in hemodialysis patients.

The purpose of this study was to evaluate the association between RDW and cerebral stroke based on the clinical data of 442 hemodialysis patients.

## Methods

### Study subjects

The China Collaborative Study on Dialysis (CCSD) is an epidemiologic survey to evaluate cardiovascular morbidity, including cerebral stroke. The CCSD was undertaken in nine of the largest dialysis facilities in six developed cities around China [4]. All patients enrolled in the CCSD were aged ≥18 years and had a dialysis vintage >3 months (three times per week for 4-h sessions). Our center is the biggest of the nine dialysis facilities and provided the largest number of hemodialysis patients (*n* = 468) between 1 January 2010 and 1 January 2012. In the CCSD, 166 patients with a medical history of CVD at dialysis were excluded from the final analysis to address the question whether dialysis affects cardiovascular risk [4]. Due to a different purpose and design, these 166 excluded patients were included in the present study. Also, 26 patients were excluded because the complete blood count was examined less often than once in every 6 months. Finally, 442 patients were included in the present study. The cohort was followed up to 30 June 2015.

### Ethics statement

In applications to the Ethical Review Committee of Guangdong General Hospital and Guangdong Academy of Medical Science (Guangdong, China), we pointed out specifically that, due to the character of a retrospective design, asking for written or verbal informed consent was not possible. Thus, the Institutional Review Board waived the normal requirement for informed consent because we used only record linkage data that have been anonymized. The waiver was obtained as permission number GDREC2016192H.

### Data collection

Baseline records were from cross-sectional data of the CCSD. The Charlson Comorbidity Score was calculated using *International Classification of Diseases, 10th Revision* (ICD-10) diagnosis codes. All patients had end-stage renal disease (ESRD), so the Charlson Comorbidity Score was increased by 2 [16].

In our center, routine medical evaluations are done in most patients every 3–6 months. Evaluated items were complete blood count, indicators for nutrition, metabolism, inflammation, anemia, mineral bone disorders, dialysis adequacy and diagnosis from other medical units. Blood samples for complete blood counts were taken before heparin administration. To avoid potential interference with RDW, the results of nonroutine complete blood count (e.g., during active bleeding) were excluded. Patients with routine medical evaluations examined less than once in every 6 months were considered to have ‘low compliance’, which is a major confounder for adverse outcomes. In addition, if the interval between RDW measurements is too long, it will affect the result, so patients without one complete blood count result within 6 months were excluded. Baseline RDW values were recorded at the beginning of the study, and the average value was the mean during the whole study period or from the beginning to the endpoint (newly diagnosed stroke). The reference interval for RDW in our center was 11–16%. In the present study, the quartile of RDW was used as the cutoff point for stratification analysis.

### Clinical definition and cohort study

‘Strokes’ were defined as a diagnosis in medical records according to ICD-10 diagnosis codes of I60-I61 for ‘hemorrhagic stroke’ or I63 for ‘ischemic stroke’. In our center, all symptomatic strokes are confirmed at least by computed tomography (CT) or magnetic resonance imaging (MRI). A symptomatic stroke was defined as an acute clinical neurologic syndrome with localizing symptoms of more than 24 h duration. All stroke events during follow-up were recorded. Newly diagnosed stroke from baseline was defined as an endpoint. Patients who died, or changed their treatment to peritoneal dialysis or renal transplantation before the study endpoint, or transferred to other hemodialysis centers, were defined as ‘censored cases’. However, fatal stroke was defined as an endpoint event rather than a censored event.

### Statistical analyses

Data with a normal distribution are given as the mean ± SD. Differences in mean values were tested using the independent-samples *t*-test. Data with a non-normal distribution are expressed as medians (25th percentile, 75th percentile). Between-group comparisons were done with a Mann–Whitney *U* test. Numerical data are described as proportions, and differences were compared with the *χ*^2^ test. The association of RDW with other variables was evaluated by Spearman’s correlation analysis. The cumulative incidence of endpoint events was calculated with the Kaplan–Meier method, and the incidence curve was plotted. The cumulative incidence curve was compared with a log-rank test. The risk factors for cerebral stroke were identified with a multivariable Cox proportional hazard model, and the variables with significance (*p* < .050) in univariate analyses were included in the multivariable Cox proportional hazard model. The risk of developing cerebral stroke was described as a hazard ratio (HR) and 95% confidence interval (CI). All statistical analyses were undertaken using SPSS v20.0 (IBM, Armonk, NY). *p* < .05 (two-tailed) was considered as significant.

## Results

### Patient characteristics

The 442 patients (53.2% male) who underwent hemodialysis had a mean age of 60.4 ± 14.3 years. None of them had severe coagulopathies. During the observation period, 10 patients (2.3%) underwent renal transplantation, 3 (0.9%) changed to peritoneal dialysis and 55 (12.4%) were transferred to other dialysis centers. Until the study endpoint, patients received a median observation of 50 (range, 27–65) months, and there were 62 newly diagnosed stroke cases (14.1%): 41 (9.3%) with cerebral infarction and 21 (4.8%) with cerebral hemorrhage. There were no significant differences in sex, urea clearance index (*Kt*/*v*), dialysis vintage, hemoglobin level or blood lipid level between cases with and without newly diagnosed stroke. However, higher age, the Charlson Comorbidity Score and incidence of hypertension, diabetes, ischemic heart disease as well as lower albumin level and indices of iron metabolism, were detected in patients with newly diagnosed stroke compared with those without newly diagnosed stroke (Table 1).

**Table 1.** Demographic and clinical characteristics at baseline.

	Nonstroke patients (*n* = 380)	Stroke patients (*n* = 62)	*p* value
Age (years)	59.6 ± 14.6	65.9 ± 10.6	.001
Number of men (%)	198 (52.1%)	37 (59.7%)	.268
Dialysis vintage (months)	15.0 (3.0, 43.5)	8.5 (3.0, 39.0)	.294
*K*t/*v*	1.57 ± 0.39	1.46 ± 0.52	.086
SBP (mmHg)	148 ± 21	153 ± 20	.094
DBP (mmHg)	78 ± 13	77 ± 13	.915
Laboratorial parameters			
ALB (g/L)	30.7 ± 4.4	29.4 ± 3.4	.030
CRP (mg/L)	3.1 (1.2, 7.0)	3.2 (2.0, 8.4)	.386
FBG (mmol/L)	6.5 ± 2.9	7.2 ± 3.6	.140
CHOL (mmol/L)	4.18 ± 1.10	4.16 ± 1.01	.895
HDL-C (mmol/L)	1.09 ± 0.41	1.12 ± 0.37	.593
LDL-C (mmol/L)	2.22 ± 0.78	2.79 ± 3.70	.264
TRIG (mmol/L)	1.51 ± 1.33	1.22 ± 0.89	.128
iPTH (pg/ml)	220 (108, 468)	199 (91, 348)	.247
Hemoglobin (g/L)	99 ± 23	95 ± 20	.186
Iron (mmol/L)	9.0 (6.5, 12.0)	8.0 (5.6, 11.2)	.081
Ferritin (μg/L)	190 (71, 438)	123 (35, 371)	.063
Transferrin (g/L)	1.62 (1.31, 2.00)	1.88 (1.50, 2.00)	.032
TSAT (%)	22.8 (15.6, 32.0)	18.4 (11.6, 26.8)	.006
Comorbidities			
Hypertension (*n*, %)	154 (40.5%)	34 (54.8%)	.035
Diabetes (*n*, %)	110 (28.9%)	29 (46.8%)	.005
Charlson Comorbidity Score	2 (2, 4)	4 (2, 5)	<.001
Ischemic heart disease (*n*, %)	46 (12.1%)	17 (27.4%)	<.001
History of stroke (*n*, %)	39 (10.3%)	14 (22.6%)	.006

SBP: systolic blood pressure; DBP: diastolic blood pressure; ALB: albumin; CRP: C-reactive protein; FBG: fasting blood glucose; CHOL: cholesterol; HDL-C: high-density lipoprotein cholesterol; LDL-C: low-density lipoprotein cholesterol; TRIG: triglyceride; iPTH: intact parathyroid hormone; TSAT: transferrin saturation.

### RDW-related factors

Baseline RDW was positively correlated with C-reactive protein (CRP) level, age, ferritin level and dialysis vintage. Baseline RDW was negatively correlated with hemoglobin level, albumin level, iron and transferrin saturation (TSAT) (Table 2). These findings suggested that RDW is correlated with anemia and iron metabolism [7], as well as inflammation and nutritional status.

**Table 2.** Correlation between RDW and other factors at baseline.

	*β*	*p* value
Age	0.164	.001
Dialysis vintage	0.144	.002
CRP	0.236	<.001
CHOL	−0.044	.377
LDL-C	−0.004	.945
Albumin	−0.181	<.001
Hemoglobin	−0.134	.005
Iron	−0.204	<.001
Ferritin	0.017	.723
TSAT	−0.146	.002
Charlson comorbidity score	0.073	.124

CRP: C-reactive protein; CHOL: cholesterol; LDL-C: low-density lipoprotein cholesterol; TSAT: transferrin saturation.

**Figure 1.**
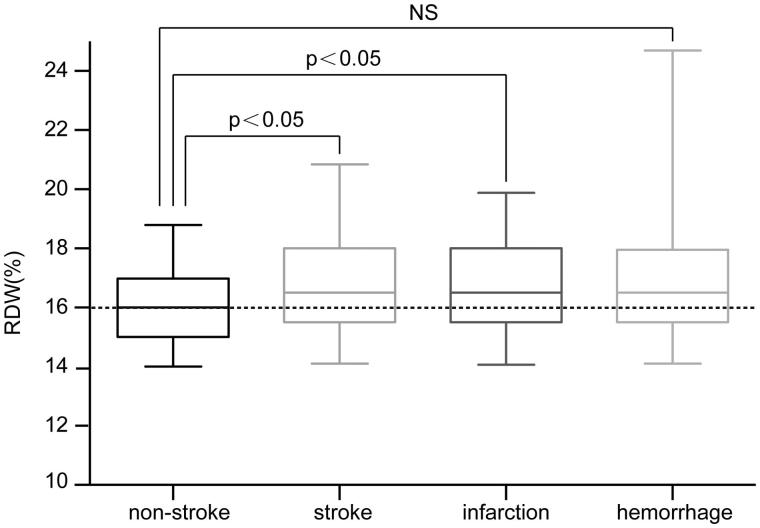
Comparison of baseline RDW between patients with cerebral stroke and nonstroke patients.

A significantly higher baseline RDW was observed in stroke patients than in nonstroke patients [16.5% (15.5%–18.0%) vs. 16.0% (15.0%–17.0%), *p* = .003]. After cerebral stroke had been classified into ‘cerebral infarction’ and ‘cerebral hemorrhage’, patients with cerebral infarction were found to have a significantly higher RDW than nonstroke patients [16.5% (15.5%–18.0%) vs. 16.0% (15.0%–17.0%), *p* = .011]. However, no significant difference was observed between cerebral-hemorrhage patients and nonstroke patients [16.5% (15.5%–18.0%) vs. 16.0% (15.0%–17.0%), *p* = .982] (Figure 1).

### Association between RDW and cerebral stroke

**Figure 2.**
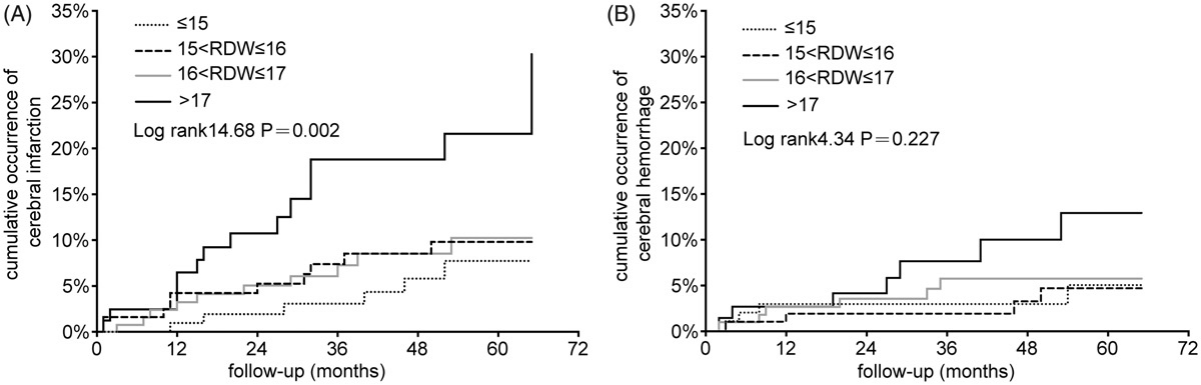
Kaplan–Meier curve of stroke incidence at graded mean RDW quartiles.

RDW fluctuates during follow-up, but the best value to reflect the overall level of RDW is not known. A recent study suggested that a single measurement may be less valuable [12]. We first analyzed the relationship between baseline RDW quartiles and incidence rate of stroke, but the result was negative despite a nonsignificant tendency for stroke (data not shown). Then, the mean of RDW was used, and the mean RDW values before stroke were used for the stroke patients. Patients with a higher mean RDW quartile had a higher incidence rate of stroke (Table 3). Therefore, we used baseline RDW for baseline description, and mean RDW during follow-up for Kaplan–Meier and Cox proportional hazard analyses. The Kaplan–Meier curve revealed a significant correlation between the high quartile of RDW and cumulative incidence of cerebral infarction (log-rank value = 14.68, *p* = .002) (Figure 2(A)), and no correlation between the RDW quartile and cerebral hemorrhage (log-rank value = 4.34, *p* = .227) (Figure 2(B)).

**Table 3.** Incidence rate of cerebral stroke in graded mean RDW quartiles.

RDW	Cerebral stroke (*n*, %)	Cerebral infarction (*n*, %)	Cerebral hemorrhage (*n*, %)
First quartile	10 (9.0%)	6 (5.4%)	4 (3.6%)
Second quartile	14 (11.4%)	10 (8.1%)	4 (3.3%)
Third quartile	16 (12.6%)	10 (7.9%)	6 (4.7%)
Fourth quartile	22 (17.0%)	15 (11.6%)	7 (5.4%)
*p* value	.002	.008	.177

Mean RDW quartile, age, hypertension, albumin level, Charlson Comorbidity Score and CRP level were identified as potential risk factors for cerebral infarction in the univariate Cox proportional hazard model. DM was the major content (accounted for 63.5%, apart from renal failure) of the Charlson Comorbidity Score in this cohort and the DM score in the Charlson system was correlated significantly with the Charlson Comorbidity Score (coefficient of Spearman’s correlation was 0.787, *p* < .001). Hence, we did not include DM in multivariable models to avoid collinearity with the Charlson Comorbidity Score. After multivariable adjustment, patients with the highest quartile of mean RDW had a 2.99-fold risk of developing cerebral stroke (HR = 2.99, 95%CI: 1.37–6.52) (Table 4) and a 3.55-fold risk of developing cerebral infarction (HR = 3.58, 95%CI: 1.35–9.51) (Table 5) compared to those with the lowest quartile of mean RDW. However, neither univariable nor multivariable Cox proportional hazard models revealed a significant association between RDW and cerebral hemorrhage (Table 6). These results demonstrated that patients with a higher quartile of mean RDW had a higher risk of developing cerebral infarction, and that mean RDW is not a risk factor for cerebral hemorrhage.

**Table 4.** Risk of cerebral stroke predicted by a multivariable cox proportional hazard model.

	Unadjusted		After adjustment^a^	
Variables	HR (95%CI)	*p* value	HR (95%CI)	*p* value
RDW		<.001		.015
First quartile	Reference		Reference	
Second quartile	1.27 (0.56–2.86)	.567	1.26 (0.54–2.92)	.620
Third quartile	1.45 (1.45–3.20)	.356	1.37 (0.59–3.16)	.465
Fourth quartile	3.60 (1.70–7.60)	.001	2.99 (1.37–6.52)	.006
Age (per one year increase)	1.04 (1.02–1.06)	<.001	1.03 (1.00–1.05)	.030
Hypertension (Yes)	1.97 (1.19–3.26)	.008	1.45 (0.85–2.50)	.175
Charlson Comorbidity Score (per 1 score increase)	1.35 (1.19–1.54)	<.001	1.24 (1.06–1.44)	.007
Duration of dialysis (per 1 month increase)	0.99 (0.99–1.00)	.996		
Hemoglobin (per 1 g/L increase)	0.99 (0.98–1.00)	.147		
C-reactive protein (per 1 mg/L increase)	1.01 (1.00–1.02)	.044	1.01 (0.99–1.02)	.243
Albumin (per 1 g/L increase)	0.92 (0.86–0.96)	.001	0.97 (0.91–1.02)	.211
Atrial fibrillation	1.79 (0.81–3.93)	.148		

a Adjusted by RDW, age, hypertension, albumin, Charlson Comorbidity Score and C-reactive protein.

**Table 5.** Risk of cerebral infarction predicted by a multivariable cox proportional hazard model.

	Unadjusted		After adjustment^a^	
Variable	HR (95%CI)	*p* value	HR (95%CI)	*p* value
RDW		.005		.027
First quartile	Reference		Reference	
Second quartile	1.53 (0.56–4.22)	.407	1.63 (0.58–4.61)	.355
Third quartile	1.54 (0.56–4.23)	.405	1.38 (0.49–3.92)	.543
Fourth quartile	4.33 (1.68–11.16)	.002	3.58 (1.35–9.51)	.012
Age (per one year increase)	1.06 (1.03–1.09)	<.001	1.05 (1.02–1.09)	.002
Hypertension (Yes)	1.73 (0.94–3.22)	.079		
Charlson Comorbidity Score (per 1 score increase)	1.47 (1.28–1.69)	<.001	1.40 (1.18–1.66)	<.001
Duration of dialysis (per 1 month increase)	1.00 (0.99–1.00)	.321		
Hemoglobin (per 1 g/L increase)	0.99 (0.98–1.01)	.340		
C-reactive protein (per 1 mg/L increase)	1.01 (1.00–1.02)	.086		
Albumin (per 1 g/L increase)	0.92 (0.87–0.98)	.007	0.98 (0.92–1.04)	.472
Atrial fibrillation	2.45 (1.03–5.82)	.043	2.56 (1.06–6.19)	.037

a Adjusted by RDW, age, albumin, Charlson Comorbidity Score and Atrial Fibrillation.

**Table 6.** Risk of cerebral hemorrhage predicted by a multivariable cox proportional hazard model.

	Unadjusted		After adjustment^a^	
Variable	HR (95%CI)	*p* value	HR (95%CI)	*p* value
RDW		.136		.145
First quartile	Reference		Reference	
Second quartile	0.92 (0.23–3.67)	.904	1.08 (0.27–4.36)	.912
Third quartile	1.39 (0.39–4.91)	.613	1.73 (0.48–6.26)	.404
Fourth quartile	3.19 (0.93–10.92)	.065	3.47 (1.01–11.92)	.049
Age (per one year increase)	1.01 (0.98–1.04)	.593		
Hypertension (Yes)	2.63 (1.09–6.36)	.032	2.56 (1.04–6.31)	.041
Charlson Comorbidity Score (per 1 score increase)	1.10 (0.82–1.47)	.546		
Duration of dialysis (per 1 month increase)	1.00 (0.99–1.01)	.696		
Hemoglobin (per 1 g/L increase)	0.99 (0.97–1.01)	.191		
C-reactive protein (per 1 mg/L increase)	1.01 (0.99–1.03)	.219		
Albumin (per 1 g/L increase)	0.92 (0.85–0.99)	.027	0.93 (0.85–1.00)	.063

a Adjusted by RDW, hypertension and albumin.

## Discussion

This single-center cohort study evaluated the association between mean RDW and newly diagnosed cerebral stroke in 442 hemodialysis patients. Our study showed higher RDW in patients with cerebral infarction than that in nonstroke patients. A multivariable Cox proportional hazard model showed a significant correlation between high RDW and newly diagnosed cerebral infarction. However, in univariable and multivariable analyses, no association was observed between RDW and cerebral hemorrhage.

The exact mechanisms underlying the association between RDW and cerebral infarction are not clear, and may be explained by several factors. Inflammatory factors and oxidative stress are important mechanisms causing cerebral infarction [17]. Also, inflammatory mediators may interfere with iron metabolism and RBC lifespan, resulting in a rise in RDW [18,19]. RDW is associated with inflammatory indices such as the erythrocyte sedimentation rate, as well as levels of CRP, interleukin-6, tumor necrosis factor receptor I and soluble transferrin receptor [18,20,21], observations that are in accordance with our findings demonstrating an association of RDW with CRP level and indices of iron metabolism. These results suggest that RDW may be a marker that indicates ‘micro-inflammation’ in hemodialysis patients. Vitamin-D3 deficiency, which is common in uremic patients, may be the other mechanism underlying the association between RDW and cerebral infarction. A reduced level of vitamin D is a potential risk factor for stroke [6,22,23]. Conversely, deficiency of active vitamin D causes disorders in the reproduction and maturation of erythrocytes, which increases the risk of anemia [6,24].

Dyslipidemia is another conventional risk factor for cerebral infarction. RDW is correlated with dyslipidemia in nondialysis patients [25]. However, we did not detect an association between RDW and low-density lipoprotein cholesterol (LDL-C) concentration in hemodialysis patients, which could be attributed to unapparent dyslipidemia in dialysis patients. Most hemodialysis patients not on lipid-lowering medications have normal or borderline-high levels of total cholesterol [26].

RDW is simple and cost-effective to measure in clinical practice. Measurement of advanced oxidation protein products (AOPPs) level is a commonly used cost-effective index of inflammation and oxidative stress in uremia, and is used widely as a marker of CVD in uremia [27]. However, AOPP level was correlated with ischemic heart disease rather than stroke markers in the CCSD study [28]. We suggest that RDW may be a supplement to uremic markers available currently, which do not represent cerebral infarction. Considering that complete blood count is the simplest and most cost-effective routine measurement in clinical practice, we think RDW is a promising marker for cerebral infarction.

Given the intrinsic limitations of all retrospective designs, other information associated with stroke was not available: cardiovascular drugs, homocysteine level, or other markers for stroke. Therefore, the association between RDW and stroke must be confirmed in further studies. Our study also had several limitations. First, stroke is a multiple-factor disease. Only some factors were corrected in the multivariable models, and prophylaxis for stroke was not considered. Second, though RDW was not associated with cerebral hemorrhage, the highest quartile of RDW had a higher risk of developing cerebral hemorrhage compared with the lowest quartile (*p* = .049). The negative results of RDW as a marker of cerebral hemorrhage may be attributed to the small sample size. However, our results demonstrated, at the very least, that RDW is not a strong marker of cerebral hemorrhage. Studies have reported that RDW is an acute-phase reactant, which suggests that the most recent RDW before stroke might be more promising than the RDW measured some time previously. However, these data are difficult to acquire because stroke cannot be predicted accurately. In our study, the negative correlation between baseline RDW and stroke might have been because of the long intervals from baseline to stroke events; whereas the high mean RDW was associated with stroke, as reported by Yoon et al. [12]. A potential reason for this association might be the cumulative role of chronic increased RDW on the cerebrovascular system. No matter which time-point was best for measuring RDW, our results indicated that an increased mean RDW was a risk factor for stroke; this merits further study in multicenter and large-sample studies.

In conclusion, the results of this study demonstrate that RDW is correlated with newly diagnosed cerebral infarction in hemodialysis patients. RDW is a promising marker of cerebral infarction.
